# Intramural esophagic hematoma secondary to coumarinic anticoagulation: a case report

**DOI:** 10.1186/1757-1626-2-9368

**Published:** 2009-12-21

**Authors:** Álvaro M Quintero, María E Gaviria, Jon K Balparda, Héctor R Cuervo

**Affiliations:** 1 Department of cardiology, Clínica Cardiovascular, Medellín, Colombia; 2 School of medicine, SIFAM Research Group, Universidad Pontificia Bolivariana, Medellín, Colombia; 3 Department of general medicine, Clínica Cardiovascular, Medellín, Colombia

## Abstract

Esophagic Intramural Hematoma is an uncommon clinical condition, with a prognosis which is essentially benign. On most cases, a predisposing or precipitating factor may be seen, with the most common ones being the history of esophagic instrumentation, food impactations and thrombocytopenia. In the following manuscript, the authors present the case of a 54-years-old male with history of valve replacement surgery, who was treated at the Clinica Cardiovascular (Medellin, Colombia), with a clinical case of Intramural Esophagic Hematoma that was later confirmed to be due to a Coumarinic overanticoagulation. On this case, it is evidenced that Intramural Esophagic Hematoma is an unrecognized complication of Courmarinic anticoagulation therapy.

## Introduction

IEH was described for the first time by Williams in 1957[1]. It is an very uncommon clinical entity[2], but fortunately its prognosis remains essentially benign with only very few patients requiring an invasive therapeutic approach[3]. Nevertheless, a high level of suspicion is required in order to make an accurate diagnosis due to the unspecific nature of its clinical presentation.

In the present article, we describe the case of a 54 year old male individual who had undergone aortic mechanical valve replacement surgery in our institution, and as a result needed lifetime anticoagulation therapy with warfarin. He presented to us approximately two and a half months after the procedure with symptoms of dysphagia and was eventually diagnosed with a symptomatic IEH secondary to supratherapeutic anticoagulation despite usual doses of warfarin and strict adherence to recommendations.

## Case presentation

A 54 year old male underwent coronary artery bypass graft (CABG) surgery and replacement of his native aortic valve for a mechanical prosthesis for treatment for severe aortic insufficiency at our institution. His postoperative course was uneventful and after initiation of warfarin therapy and careful monitoring of his Intrenational Normatized Ratio (INR) to ensure therapeutic anticoagulation (target INR 2.5-3.5), the patient was discharged home to continue ambulatory warfarin therapy at a dose of 5 mg daily.

48 days after surgery, the patient was readmitted to our institution because of severe pain in his left flank. During this hospitalization he was diagnosed with a large splenic hematoma and an associated massive hemoperitoneum, his INR at admission was 9.4. He was initially managed conservatively, but after two days it was decided that the patient required invasive treatment with splenectomy and drainage of the hemoperitoneum because of a drop of 4 gr/dl in hemoblobin value within a day. At discharge the warafrin dose was readjusted to 2.5 mg with an INR of 2.8.

His second postoperative course was uncomplicated until 25 days later (73 days after the initial valve replacement surgery), when he presented to our Emergency Department with a 24 hour history of frank dysphagia and associated dyspepsia and flatulence.

Upon initial evaluation he was noted to be hemodynamically stable with a blood pressure of 122/76 mmHg and a heart rate of 65 beats per minute and he was on beta blocker therapy. Upon further evaluation, a mildly bleeding, vesicular lesion has found on his right oropharynx; there was not tenderness to neck palpation or regional lymphadenopathy. The patient denied a history of obvious spontaneous bleeding or painful swallowing. Laboratory tests ordered at that time revealed supratherapeutic anticoagulation with an INR "infinite", and a hemoglobin and hematocrit levels of 10.1 gr/dL and 31,4% respectively (Table 1. Other laboratory tests included the following: Leukocytes 11.610, Neutrophil 76%, serum creatinine 0,9 mg/dL, blood ureic nitrogen (BUN) 14 mg/dL. Warfarin therapy was immediately discontinued in an attempt to normalize his clotting parameters.

**Table 1:** Relevant INR values during hospitalization.

Day of Hosp.	INR	Comments
1	"Infinite"	Initial INR; longer than 120. Warfarin was suspended.

2	"Infinite"	Longer than 120. Patient develops hematuria. SDE diagnoses IEH.

3	"Infinite"	Longer than 120. 4 units of FFP are given.

4	6.2	3 units of FFP are given.

5	5.6	4 units of FFP are given.

6	7.0	-

7	7.7	First dose of Vitamin K 10 mg is administered.

8	1.5	Hematuria resolves.

10	2.4	-

12	3.6	Second dose of Vitamin K 10 mg is administered.

16	1.9	-

19	2.2	-

21	1.8	-

23	1.5	Warfarin 2.5 mg oral is started again.

26	2.0	Normal SDE.

29	2.7	-

31	2.5	Patient is discharged.

INR = International Normalized Ratio. SDE = Superior Digestive Endoscopy. IEH = Intramural Esophagic Hematoma. FFP = Fresh Frozen Plasma.

**Figure 1:**
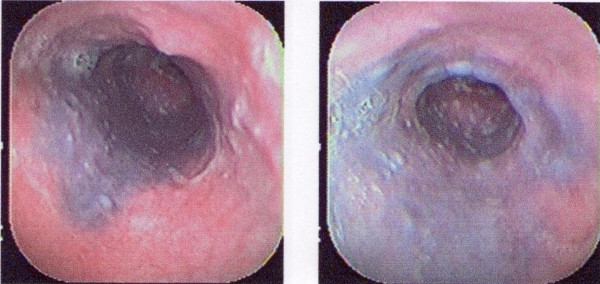
**Superior Digestive Endoscopy showing the presence of great submucosal ecchymosis compatible with Intramural Esophagic Hematoma**. Courtesy of William Darío Lopera Lotero, MD.

The initial differential diagnosis included acute pharyngitis and azitromycin was prescribed. However, due to a persistent complaint of dysphagia, a diagnostic upper endoscopy (EGD) was performed. The EGD revealed a pale distal mucosa and the presence of a large submucosal ecchymosis/hematoma that extended to the level of the cardias. These findings were consistent with an IEH (Figure 1).

During the course of his hospitalization, in addition to his diagnosis of IEH, our patient had other clinical and laboratory manifestations that reflected his supratherapeutic anticoagulation status, including macroscopic hematuria and severe worsening anemia. He received multiple transfusions of fresh frozen plasma (FFP) and packed red blood cells (PRBC), as well as administration of vitamin K. His coagulation parameters eventually returned to normal and after an observation period of ten days, anticoagulation with warfarin was restarted with careful serial monitoring of his INR, remaining within therapeutic level throughout the rest of his hospital stay and without any bleeding complications.

**Figure 2:**
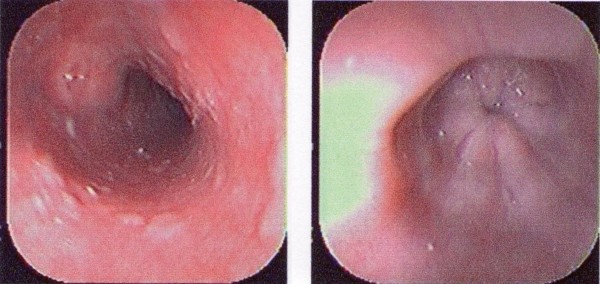
**Superior Digestive Endoscopy showing the absence of submucosal ecchymosis, compatible with spontaneous resolution of Esophagic Hematoma**. Courtesy of William Darío Lopera Lotero, MD.

Five days before discharge from the hospital, an EGD was performed, which showed no evidence of the findings of the previous EGD, performed 24 days earlier, suggesting a spontaneous resolution of the IEH (Figure 2). After discharge, the patient had a close clinical follow up, and his INR was monitored at frequent intervals. His symptoms improved completely and he has had no further clinical or laboratory evidence of coumarinic toxicity.

## Discussion

IEH is an uncommon, although well documented cause of acute thoracic pain[4], often associated with additional digestive symptoms such as dysphagia and hematemesis[5,6]. The condition has been described to occur spontaneously as consequence of an abrupt rise on intraesophageal pressure[7,8], although in most cases, a precipitating event may be found, the most common being a history of previous esophageal instrumentation[5].

According to Martínez et al[5], the most common clinical manifestation among patients with IEH is acute thoracic pain in 87% of cases, followed by hematemesis, dysphagia and pain while swallowing in 70%, 26% and 26% of cases respectively. It is important to mention that in up to 60% of cases there is a history of a predisposing factor, most commonly a history of esophageal instrumentation, food impaction and bleeding dyscrasias like thrombocytopenia[5,9,10]. Well documented cases of IEH secondary to anticoagulation therapy with warfarin are sparse in worldwide literature, which makes the present report all the more relevant[7,11,12].

The case we have just presented is, to the author's knowledge, the first reported case of IEH secondary to coumarinic anticoagulation reported from a Latin American institution and one of the few reported from around the world[7,11,12]. Coumarinics, in particular warfarin, are medicines prescribed frequently for a myriad of medical conditions and in all sorts of patients. The practicing physician should be aware of the potential complications of anticoagulation, including IEH, a condition that albeit uncommon, may carry a lethality of up to 7-9% in patients with other associated commorbidities[7].

In this case, it is fairly clear that this rare complication was the result of a supratherapeutic level of anticoagulation with warfarin, medication which was clinically indicated after the patient's CABG and valve replacement surgery. Upon presentation to our emergency department, this individual had an INR reported as >120 seconds, otherwise known as an "infinite INR", reflecting the laboratory's inability to accurately calculate it. Although there was no history of immediate active bleeding prior to presentation, our patient had recently been hospitalized with a splenic hematoma and hemoperitoneum that required surgical intervention and drainage, and at this time his INR level was found to be elevated above the therapeutic range. This severe clotting dysfunction may be enough to justify spontaneous bleeding on multiple sites of the human body, including those that may not be evident to a careful physical examination.

This clinical case also highlights the benign course of IEH[3,4,13], as the condition resolved spontaneously once clotting mechanisms are normalized. It must be stressed however that its benign course is dependent on the consideration of this entity in the differential diagnosis and work up of a patient who presents with symptoms and signs compatible with IEH and who has a predisposing condition making him prone to developing it, hence stressing the importance of this report.

## Consent

Written informed consent was obtained from the patient for publication of this case report and accompanying images. A copy of the written consent is available for review by the Editor-in-Chief of this journal.

## Competing interests

The authors declare that they have no competing interests.

## Authors' contributions

AMQ and HRC were involved in the medical treatment of the patient, MEG and JKB reviewed the patient's medical chart and wrote the initial draft for the article. All authors were involved in writing and reviewing the article. All authors have read and approved the final version of the manuscript.
